# An Update on Appendiceal Neuroendocrine Tumors

**DOI:** 10.1007/s11864-023-01093-0

**Published:** 2023-05-04

**Authors:** Elisa Andrini, Giuseppe Lamberti, Laura Alberici, Claudio Ricci, Davide Campana

**Affiliations:** 1 Department of Experimental, Diagnostic & Specialty Medicine (DIMES), University of Bologna, 40138 Bologna, Italy; 2 Medical Oncology, IRCCS Azienda Ospedaliero-Universitaria Di Bologna, Via P. Albertoni, 15, 40138 Bologna, Italy; 3 Department of Internal Medicine and Surgery (DIMEC), Alma Mater Studiorum, S. Orsola-Malpighi Hospital, University of Bologna, Policlinico S. Orsola-Malpighi Via Massarenti N. 9, 40138 Bologna, Italy; 4 Division of Pancreatic Surgery, IRCCS, Azienda Ospedaliero Universitaria Di Bologna, 40138 Bologna, Italy

**Keywords:** Appendiceal tumor, aNEN, WHO, Right-side hemicolectomy, Size

## Abstract

The mainstay of appendiceal neuroendocrine neoplasm (aNEN) treatment is surgery, based on simple appendectomy or right-sided hemicolectomy with lymphadenectomy (RHC). The majority of aNENs are adequately treated with appendectomy, but current guidelines have poor accuracy in terms of selecting patients requiring RHC, especially in aNENs 1–2 cm in size. Simple appendectomy is curative for appendiceal NETs (G1–G2) < 1 cm (if the resection status is R0), whereas RHC with lymph node dissection is recommended in tumors ≥ 2 cm in diameter, based on the high risk of nodal metastases in these cases. The clinical management of aNENs 1–2 cm in size is more controversial because lymph node or distant metastases are uncommon but possible. In our opinion, patients with tumor size > 15 mm or with grading G2 (according to WHO 2010) and/or lympho-vascular invasion should be referred for radicalization with RHC. However, decision-making in these cases should include discussion within a multidisciplinary tumor board at referral centers with the aim of offering each patient a tailored treatment, also considering that relatively young patients with long-life expectancy represent the majority of cases.

## Introduction

Appendiceal neuroendocrine neoplasms (aNENs) are relatively frequent gastrointestinal neuroendocrine tumors, with an approximate annual incidence of 0.15 to 0.6/100,000 inhabitants, a peak in young patients (mean age at diagnosis between 38 and 51 years), and a female preponderance (2:1) in some Western series [1–4].

NENs of the appendix are the largest subgroup of appendiceal tumors (~ 30–80%) and are classified, according to the 2019 World Health Organization (WHO) in well-differentiated neuroendocrine tumors (NETs), the most frequent type and the exceedingly rare poorly differentiated neuroendocrine carcinomas (NECs) and mixed neuroendocrine/non-neuroendocrineneoplasms (MiNENs) [5]. In addition, goblet cell adenocarcinoma, once classified as a mixed form of aNENs, now included into the group of adenocarcinomas, is considered a separate entity and will not be discussed in this review.

With the term “aNENs,” we will refer to sporadic, non-functioning well-differentiated tumors arising from the appendix, which are generally characterized by an indolent behavior and favorable long-term outcomes [2, 6].

aNENs are usually grade 1 (G1) or grade 2 (G2) NETs according to the 2010 WHO classification (i.e., Ki-67 index < 20%) and more than 95% of them are smaller than 2 cm in diameter [7]. Historically, aNENs were considered indolent tumors. However, a highly variable percentage of lymph node involvement at diagnosis is reported in literature (i.e., between 11 and 49% of cases), and up to 10% of cases may present with distant metastases [2, 8–10].

The majority of aNENs are adequately treated with appendectomy, but current guidelines have poor accuracy in terms of selecting patients requiring right-side hemicolectomy (RHC), especially in aNENs 1–2 cm in size.

Clinical management of aNENs is currently controversial due to the impossibility to run prospective trial and to produce definitive evidence, so that there is an urgent need for a better stratification of patients to define the optimal management of this disease. The aim of this review is to collect current clinical and pathological data regarding aNENs and emerging treatment strategies in order to guide physicians in the management of this rare disease in clinical practice.

## Clinical presentation and staging

aNENs represent up to 85% of all appendiceal neoplasms and the diagnosis is nearly always incidental during appendectomy performed for other reasons (i.e., suspected or manifest acute appendicitis or other abdominal surgeries) with a rate of approximately 3–5/1000 appendectomies [1, 11, 12]. Considering the incidental nature of their diagnosis, aNENs are rarely symptomatic in the large majority of cases. Indeed, the more frequent location of aNENs is in the tip of the appendix (~ 70%), whereas only 5–20% of cases arise in the mid-appendix and < 10% of cases in the base, so that rarely cause appendix obstruction [13]. The association with hormone production causing carcinoid syndrome is extremely rare (less than 1% of cases) and only occurs in presence of metastatic disease [14].

Regarding aNENs staging, the American Joint Committee on Cancer (AJCC)/Union for International Cancer Control (UICC) and the European Neuroendocrine Tumor Society (ENETS) classifications differ in the definitions of T stages (Table 1) [11, 15].

**Table 1:** Comparison of ENETs and UICC/AJCC classification according to T stage

	*ENETS guidelines*	*UICC/AJCC classification*
*T1*	≤ 1 cm with infiltration of the submucosa and muscularis propria	T1a, ≤ 1 cm T1b, > 1 but ≤ 2 cm
*T2*	≤ 2 cm with infiltration of the submucosa, muscularis propria, and/or minimal (≤ 3 mm) invasion of mesoappendix	> 2 but ≤ 4 cm or invasion of cecum
*T3*	> 2 cm and/or > 3 mm invasion of subserosa/mesoappendix	> 4 cm or invasion of ileum
*T4*	Invasion of peritoneum/other organs	Perforation of peritoneum or direct invasion of adjacent organs or structures

According to the National Comprehensive Cancer Network (NCCN) guidelines, patients with tumor ≥ 2 cm or any tumor size with incomplete resection or positive nodes should undergo abdominal/pelvic multiphasic computed tomography (CT) or magnetic resonance imaging (MRI) to rule out locoregional or distant metastases [16]. Indeed, lymph node metastases are reported in about 2.5% of tumors < 1 cm, in 31% of tumors > 1 cm but < 2 cm, and in 64% of tumors ≥ 2 cm [17].

## Treatment

The mainstay of aNEN treatment is surgery, based on simple appendectomy or right-sided hemicolectomy with lymphadenectomy (RHC). The incidental discovery of an aNEN during appendectomy performed for other reasons generates a clinical challenge to select the cases in which the simple appendectomy is sufficient or those that need a second surgical look with RHC, in order to obtain a radical lymph node dissection and to prevent the risk of recurrence [18, 19]. Both the North American Neuroendocrine Society (NANETS) and the ENETS guidelines suggest a tailored approach: simple appendectomy is considered curative for appendiceal NETs (G1–G2) < 1 cm (if the resection status is R0), whereas RHC with lymph node dissection is recommended in tumors ≥ 2 cm in diameter (T3 according to ENETS, T2 according to UICC/AJCC), based on the high risk of nodal metastases in these cases [11, 20]. The clinical management of a T2 (ENETS) or T1b (UICC/AJCC) NET (i.e., tumors with diameters > 1 cm but < 2 cm) is more controversial because lymph node or distant metastases are uncommon but possible, so definitive cure can be achieved with a RHC. However, this procedure is burdened by an increased peri- and post-operative morbidity compared to a simple appendectomy, so each case should be accurately evaluated, also considering the relatively young age of patients and the consequent long-life expectancy, and avoid over-treatment. Thus, additional risk factors should be taken into consideration, such as the localization of the tumor at the base of the appendix (particularly with R1 resection), a mesoappendiceal infiltration > 3 mm, G2 tumors (i.e., Ki-67 index of ≥ 3%), and lympho-vascular invasion. In case of coexistence of one or more of these criteria, RHC should be discussed with the patient, although no data on survival benefit of a more aggressive surgery are currently available. Indeed, current guidelines are predominantly based on small, single-institution case series and retrospective data, whereas prospective studies are not feasible and thus lacking. As a consequence, these additional criteria for radicalization in patients with aNENs cannot be prospectively validated, due to the relatively indolent course and the rarity of aNEN that makes such studies difficult to power, and even retrospective analyses are limited by small sample size. Based on these data, there is a diffuse perception that current guidelines are inadequate, especially for the “gray zone” of aNENs between 1 and 2 cm, in which the debate is still underway whether RHC constitutes an over-treatment or is oncologically adequate. For example, a retrospective series of 28 consecutive aNEN patients who underwent RHC has reported that the use of ENETS criteria for RHC could leave residual disease in 18% of cases [21]. Nevertheless, to date there is no data regarding the long-term effect of residual disease on survival outcomes.

## Prognostic factors

### Size

The size of a primary tumor is certainly considered the most relevant predictive factor for nodal involvement and, consequently, the most powerful indicator for deciding the extent of surgery. However, the best cut-off value has been a matter of debate over the last decades. Two retrospective studies in unselected patients with aNEN found that the 2 cm cut-off was the only risk factor of nodal involvement, justifying a more aggressive surgery in these patients [22, 23].

Indeed, 30% (*N* = 7/23) of aNENs ≥ 2 cm developed metastases whereas no metastases were observed in tumors < 2 cm (*N* = 127) [23]. However, in both studies, the majority of cases of aNENs had a diameter < 1 cm, whereas only a small percentage of cases had a diameter > 2 cm (*N* = 3 and *N* = 23, respectively) [22, 23].

A population-based analysis of 576 patients with aNENs from the Surveillance Epidemiology and End Results (SEER) showed a significant association between tumors size ≥ 2 cm and the incidence of lymph node metastases (40.6%) [9]. However, this study had several limitations, including the use of a registry that did not collect information about several known prognostic factors in aNENs (e.g., tumor grading) and the missing tumor size in a significant proportion of patients (47%).

Although there is an overall consensus about the high rate of nodal disease in aNENs ≥ 2 cm in size, the management of tumors 1–2 cm is more controversial. In fact, in 89 cases of aNENs from the SEER a higher than expected rate of lymph node metastases in 15%, 47%, and 86% of tumors < 1 cm, 1–2 cm, and > 2 cm in size, respectively, has been reported [2]. Moreover, in a small series of 12 patients, residual disease after appendectomy was present in 3 of 12 (25%) patients who underwent RHC and had a tumor size < 2 cm [24]. In a retrospective series of 28 consecutive patients with aNENs who underwent RHC at three different tertiary centers, among 13 patients (46%) with tumor size between ≥ 1 and < 2 cm, a residual disease after RHC was present in 5 of 28 patients (18%), and among 5 of 13 patients (38%) with this tumor size, lymph node metastases were found despite the mesoappendiceal invasion < 3 mm [21]. Based on these data, tumors with diameters ≥ 1 cm but < 2 cm remain a “gray zone,” whose clinical management is still controversial and under debate.

Since 1985, Anderson and Wilson suggested that a diameter > 15 mm could be a better factor of stratification of patients who should go to RHC, considering the increased risk of nodal involvement [25]. A recent large multicentric retrospective series of 435 aNEN patients from NEN-specialized centers, of whom 69 underwent RHC, analyzed factors associated with nodal involvement [26]. In this study, tumor size, grading G2 (according to WHO 2010), and lympho-vascular invasion were independent predictive factors for nodal involvement (identified in 30% of cases), whereas mesoappendiceal invasion and localization of tumor within appendix were not. Moreover, in multivariate analysis only tumor size > 15.5 mm was independently related to nodal involvement after RHC (sensitivity 0.71, specificity 0.75), suggesting that this cut-off size could be a better predictive factor of nodal involvement.

Furthermore, a recent meta-analysis of 6 studies including a total of 261 patients with aNENs who underwent complete RHC showed that a 15-mm cut-off had a similar outcome in terms of reducing unnecessary RHCs compared to a 20-mm cut-off [19].

Beyond tumor size, the other pathological features that should be taken into account in therapeutic planning include lympho-vascular invasion, grading G2, mesoappendiceal infiltration > 3 mm, and tumor location at the base of the appendix.

### Lympho-vascular invasion

Since 1995, lympho-vascular invasion has been considered a factor associated with worse prognosis [27]. A retrospective study including 263 cases of aNENs, of whom 72 underwent complete RHC, reported that vascular and lymph vessel invasion were independent risk factors for lymph node involvement at RHC [28]. A French multicenter retrospective study evaluated 403 patients with non-metastatic aNENs, of which 80 underwent RHC but overall 100 of them had lymphadenectomy [29]. The study showed that tumor size, lympho-vascular invasion, perineural invasion, and TNM stage were significantly associated with nodal involvement at RHC. However, it should be noted that lympho-vascular invasion is frequently overestimated due to artifacts during sample preparation [30]. In order to reduce this potential bias, NET-expert pathologists should assess samples or revise unclear cases [31]. Furthermore, a meta-analysis of 11 studies including a total of 602 aNEN patients who underwent RHC showed that vascular invasion and lymph vessel invasion together with tumor size and perineural invasion are strong predictors for lymph node involvement [32].

### Grading

Although tumor grading is a well-known prognostic factor in gastro-entero-pancreatic NENs, studies showing its role in aNENs are currently lacking. A retrospective review including 51 patients who underwent surgery for aNENs reported that the Ki-67 was associated with decreased survival but did not correlate with tumor size, depth of invasion, or presentation with metastatic disease [33]. By contrast, in a retrospective analysis of 138 cases, pT stages but neither mitotic count nor proliferative index were associated with worse prognosis [7]. Despite available evidence is somehow controversial, G2 grading according to WHO is considered a risk factor for the presence of nodal metastases. Indeed, the abovementioned large multicentric retrospective series showed that grading G2 is independently related to the presence of nodal involvement both at univariate and multivariate analysis [26].

### Mesoappendiceal infiltration

According to ENETS guidelines, mesoappendiceal infiltration > 3 mm is used for the characterization of T2 and T3 tumors and represents a “minor criterion” to select patients who should be proposed radicalization with RHC [11]. However, evidence about the prognostic role of mesoappendiceal infiltration are limited and several studies did not find a significant association with nodal disease and prognosis [7, 26, 30, 34]. By contrast, only the previously cited study showed a significant association of subserosa and mesoappendix invasion with poor clinical outcomes [7].

### Tumor location

Another additional parameter commonly used to refer patients to RHC is tumor location at the base of the appendix, found in about 7–10% of cases [35]. Indeed, several studies showed that aNENs located at the base of the appendix are more likely to be associated with incomplete resection and consequent increased risk of local recurrence and metastases [23, 36]. Therefore, a complete RHC is recommended for the risk of incomplete resection rather than for the tumor localization itself [37].

## When to perform a RHC?

As aforementioned, current guidelines recommended to perform a complete RHC in patients with aNENs > 2 cm or with aNENs < 2 cm with additional risk factors. However, to date conflicting data are reported in literature and there is even more awareness that the strict adherence of guidelines could lead to perform unnecessary RHCs. Indeed, among 3198 cases of aNENs from the National Cancer Database, 32.4% with tumor smaller than 2 cm underwent RHC and 31.5% with tumor larger than 2 cm received simple appendectomy only, with similar survival outcomes [38]. Because RHC is potentially associated with complications and adverse consequence in terms of long-term health-related quality of life (HRQoL) impairment, a multicenter study from five ENETS centers of excellence evaluated HRQoL outcomes and their possible association with the type of surgery [39]. Among 166 aNEN patients, 108 underwent simple appendectomy and 58 underwent RHC, of whom 38 (65.5%) did not have residual disease or lymph node involvement. Notably, among 79 patients participating to HRQoL assessment, global HRQoL was similar between the two groups of patients but impaired social functioning, diarrhea, and financial difficulties were more frequently reported in the RHC group.

**Fig. 1:**
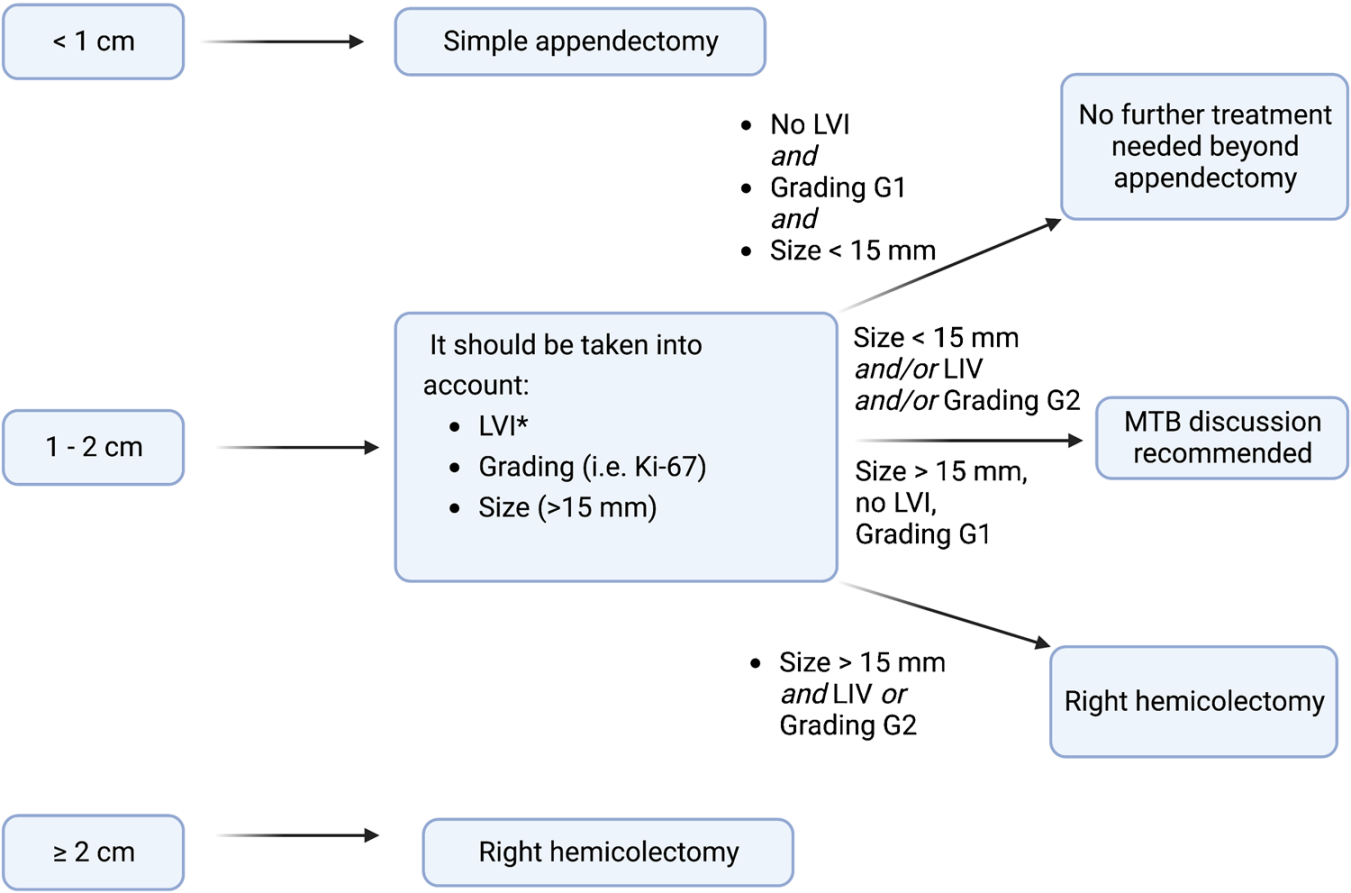
Proposed algorithm for the management of aNENs. *NET-expert pathologists should assess samples or revise unclear cases. MTB, multidisciplinary tumor board.

Based on the available data, we assume that a selection of patients based on tumor size could be helpful to reduce over-treatment. Thus, both 20-mm and 15-mm cut-offs showed a similar performance in reducing the risk of unnecessary RHC, but the 15-mm cut-off is more helpful in the evaluation of patients with aNENs 1–2 cm [19]. The other factors that should be taken into account are grading G2 and lympho-vascular invasion, which were independent predictive factors for nodal involvement [26, 29, 40, 41]. However, considering the risk of lympho-vascular invasion overestimation due to preparation artifacts, an assessment by a NET-expert pathologist would be advisable [31]. In our opinion, patients presenting tumor size > 15 mm, grading G2 (according to WHO 2010), and/or lympho-vascular invasion should be referred for radicalization with RHC. Figure 1 summarizes the proposed algorithm for aNEN management.

## Lymph node involvement: does it impact survival?

The biological significance of lymph node involvement in aNENs is currently uncertain. Several studies had not shown a significant effect of nodal metastatic involvement on outcomes of patients with aNENs [26, 29, 32, 41]. In most abdominal cancers, an oncologically radical surgery also includes lymphadenectomy, which is routinely performed together with resection of primary tumor in order to identify patients who could benefit from adjuvant chemotherapy. There is evidence that regional lymph node metastases did not have a significant impact on overall survival (OS), regardless of tumor size [2], and that aNENs with lymph node involvement had an excellent prognosis irrespective of the type of surgery, questioning the hypothesis that RHC should not improve outcome in these patients [18]. Thus, there is no data supporting that the presence of lymph node involvement affects survival outcomes and not even that RHC plays a prophylactic role. Although this could be explained by the indolent nature of aNEN which might make lymph node involvement relatively irrelevant, aNENs are often diagnosed in young patients with a long-life expectancy and follow-up time of available studies, despite spans up to 10 to 15 years in some reports, is not always sufficient, making further and larger studies needed to clarify this issue.

## Survival

Several studies, including national registry studies, retrospective cohort studies, and meta-analyses, have shown that aNENs have generally a favorable prognosis after resection, regardless of the type of resection (i.e., RHC or appendectomy alone). Indeed, the survival benefit of completing RHC compared to appendectomy is currently uncertain. A retrospective analysis of SEER data did not demonstrate a significant difference in 10-year OS rates between patients who underwent RHC compared to those who underwent appendectomy alone (72% vs 82%, respectively) [9].

The aforementioned meta-analysis, which confirmed that tumor size > 20 mm, as well as > 10 mm in combination with lympho-vascular and perineural invasion, is associated with increased risk of lymph node involvement, showed that the presence of lymph node metastases did not affect OS in patients who underwent curative resection (either RHC or appendectomy) [32]. Indeed, the 10-year disease-specific survival (DSS) rate was 99.2% among adult patients without lymph node metastases compared to 95.6% among those with lymph node involvement. In addition, a retrospective study of aNEN patients undergoing appendectomy at 3 tertiary referral centers evaluated the management of aNENs and analyzed disease recurrence and OS outcomes [42]. Among 215 patients who underwent appendectomy, 64 had indication for RHC (according to ENETS criteria) but only 49 underwent RHC, whereas 15 did not for several reasons (e.g., patient refusal, significant comorbidities). Among 49 patients who underwent RHC, 12 (24.1%) had disease recurrence whereas no death was registered at a median follow-up of 38 months. Interestingly, none of the patients who had the indication for RHC but who did not perform it developed local or distant disease recurrence, or died. Five-year and 10-year OS for all patients with aNENs were both 99.05%. In agreement with these data, a registry-based study of patients with aNENs and tumor size > 2 cm from the SEER database investigated whether RHC confers a survival advantage compared with appendectomy alone [43]. After a propensity score model matching (1:1 ratio), 109 patients undergoing appendectomy and 109 undergoing RHC were analyzed: type of intervention did not impact significantly on OS in patients with aNENs > 2 cm.

Another large, single-center retrospective study including 102 patients with aNENs evaluated the impact of lymph node involvement and RHC on survival outcomes [41]. Among 34 patients who were proposed for completing RHC based on one or more risk factors according to ENETS criteria, 4 refused surgery and none of them relapsed during 13 years of follow-up. Moreover, RHC was not associated with complications or mortality. Residual disease was noted in 9/30 patients (8 with lymph node involvement, 1 with residual tumor in caecum) that was associated with a primary aNEN size ≥ 2 cm. In the overall study population, 5-year and 10-year OS were 99% and 92%, respectively, whereas 5-year and 10-year relapse-free survival were 98% and 92%, respectively. Only one patient developed recurrence after 16.5 years of follow-up and only 5-year relapse-free survival was affected by ENETS stage. Also, a registry study conducted in Switzerland showed that the 10-year relative survival rate after resection of aNEN did not significantly differ from that of the average national population matched by age and sex [44]. More recently, a multicenter, international, retrospective, cohort study compared the role of appendectomy and RHC among 278 patients with aNENs between 1 and 2 cm in size, from 40 European institutions [45]. The study included 163 (59%) patients who underwent appendectomy and 115 (41%) who underwent RHC and showed that OS after a median follow-up of 13 months was similar between the two groups, also in multivariable Cox regression analysis including ENETS criteria. According to these data, the authors concluded that RHC is not recommended in patients with aNEN of 1–2 cm because the risk of post-operative complications and mortality overcome the potential benefits. However, results from this study should be interpreted with caution, considering the relatively short follow-up period compared to the 50-year life expectancy of young patients and the absence of a centralized histopathological review of all cases that introduces an evaluation bias. Moreover, the estimated rate of nodal metastases in the appendectomy group was 12.8% but it is not specified which factors were used and the study did not report any data about the cut-off size of 15 mm, as previously reported in literature [19].

Furthermore, the SurvivApp study, a retrospective, observational study, evaluated the frequency of distant metastases and clinically relevant relapse and mortality of aNENs 1–2 cm in size (NCT03852693). The investigators proposed that RHC had no impact on long-term survival after complete resection of aNENs 1–2 cm, assuming that the risk of recurrence is lower compared to that of oncological RHC. The study has completed enrollment and results are awaited.

In conclusion, the benefit of RHC on long-term outcomes remains controversial. The rationale for RHC is to improve survival outcomes by removing locoregional lymph nodes and reducing the risk of disease recurrence or metastases. However, data demonstrating survival benefit of completing RHC are currently lacking and, considering the indolent nature of aNENs, appendectomy could be a viable treatment option, even for tumor > 2 cm, particularly in elderly patients with significant comorbidities, who may not tolerate RHC.

Table 2 summarizes the main studies on aNEN patients and data about prognostic factors and survival.

**Table 2:** Summary of major studies on appendiceal neuroendocrine neoplasms

First author (year, journal)	Study design	*N*	No of patients undergoing RHC (*N* + , %)	Prognostic factors associated with nodal involvement	Survival in patients with *vs* without nodal involvement	Follow-up time (median)
Anderson (1985, *Br. J. Surgery*) [25]	Single-center retrospective	147	NA	Size > 1.5 cm	Not reported	Average 7.4 years (range 2–14 years)
Moertel (1987, *N Eng J Med*) [23]	Single-center retrospective	150	NA	Size ≥ 2 cm	Not reported	28 years
Groth (2011, *J Surg Oncol*) [9]	Registry-based (SEER)	576	304 (73, 27.7%)	Size ≥ 2 cm	10-year OS rates 70.6% *vs* 92.5%	≥ 10 years
Mullen (2011, *J Surg Oncol*) [2]	Registry-based (SEER)	89	NA	Size ≥ 2 cm Size 1–2 cm, young age, with MAI	10-year OS rates 90% vs 100%	Not reported
Grozinsky-Glasberg (2013, *Neuroendocrinology*) [21]	Multicenter retrospective cohort study	28	28 (10, 35.7%)	Size ≥ 2 cm Size 1–2 cm, if ≥ 1 risk factors (MAI, LVI)	Not reported	3.6 years
Steffen (2015, *World J Surg*) [44]	Multicenter retrospective	79	6 (1, 16.6%)	Second primary malignancies	10-year OS rates 96% (all patients)	13.7 years
Rault-Petit (2018, *Ann of Surg*) [29]	National registry-based	403	80 (17, 23%)	Size > 1.95 cm LVI PNI pT according to TNM	Not reported	Median 3 months (range 0–84)
Pawa (2018, *Neuroendocrinology*) [42]	Multicenter retrospective	215	49 (12, 24.5%)	No specific finding, RHC might present an over-treatment	10-year OS rates 99.05% for all patients	Median 38.5 months (range 1–143)
Galanopoulos (2019, *Neuroendocrinology*) [28]	Single-center retrospective	263	72 (23, 32%)	Grading G2 LVI	Not reported	10 years
Brighi (2020, *Ann of Surg*) [10]	Multicenter retrospective study	435	69 (21, 30.4%)	Size > 15.5 mm LVI Grading G2	DSS 74 *vs* 141 months	10 years

*RHC*, right-side hemicolectomy; *NA*, not applicable; *MAI*, mesoappendiceal invasion; *LVI*, lympho-vascular invasion; *PNI*, perineural invasion; *OS*, overall survival; *DSS*, disease-specific survival

## Follow-up

Follow-up strategy depends on the type of surgery and the features of the definitive histological examination: aNENs < 1 cm completely resected with simple appendectomy (i.e., R0) do not require specific follow-up as well as patients with tumors > 1 cm who underwent RHC without evidence of lymph nodes involvement or other residual disease [11]. For patients with tumors between 1 and 2 cm that did not received RHC for several reasons (e.g., comorbidity, patient refusal) but with risk factors (i.e., localization at the base of the appendix, mesoappendiceal invasion > 3 mm, G2 NET, or lympho-vascular invasion), ENETS guidelines recommend regular follow-up, considering the not-negligible risk to develop lymph node metastases. However, to date there are no data on the benefit of follow-up in preventing cancer recurrence or on long-term outcomes in these patients. Conversely, patients with tumors > 2 cm, with lymph node involvement or resected distant metastases or with additional risk factors (i.e., R1 resection), should undergo long-term follow-up every 3 to 6 months in the first year after resection and then every 6 to 12 months. According to ENETS guidelines, neither biochemical marker (i.e., CgA or 5-HIAA) nor non-invasive imaging is currently validated in long-term follow-up of aNENs. Considering the risk of cumulative exposure of radiation in young patients, ultrasonography and/or magnetic resonance imaging (MRI) should be preferred over computed tomography (CT) scan, whereas in older patients with high risk or distant metastases, CT or MRI is recommended, possibly in association with somatostatin receptor imaging if a disease recurrence is suspected [46, 47]. However, the potential of recurrence of this slow-growing disease should be taken into account and follow-up in aNENs > 2 cm or > 1 cm with risk factors should be life-long [48].

## Conclusions

This review summarizes all available evidence on aNENs, with the aim to guide physicians in the management of this rare disease. Though the mortality of aNENs is low, the morbidity and the impact on quality of life of surgery are substantial. According to current guidelines, patients with aNENs > 2 cm should undergo complete RHC considering the greater risk of lymph node involvement, whereas for patients with aNENs < 1 cm, simple appendectomy is curative.

To date, there is a lack of consensus regarding the management of aNENs with 1–2 cm of diameter. Based on available data in literature, in our opinion patients with tumor size > 15 mm or with grading G2 (according to WHO 2010) and/or lympho-vascular invasion should be referred for radicalization with RHC. However, decision-making in these cases should include discussion within a multidisciplinary tumor board at referral centers with the aim of offering each patient a tailored treatment, also considering that relatively young patients with long-life expectancy represent the majority of cases.

Because prospective studies though necessary are hardly feasible, clarification of the role of complete RHC and association of lymph node involvement with survival should rely on careful evaluation of well-conducted retrospective series, in order to improve the clinical management of these patients.
